# Authors' reply

**DOI:** 10.4103/0253-7613.45158

**Published:** 2008

**Authors:** Gurudas Khilnani

**Affiliations:** Department of Pharmacology, RNT Medical College, Udaipur-313 001, India. E-mail: drgurudas@rediffmail.com

Sir,

I thank you for your keen interest and comments[1] on the correspondence, ‘The concept of personal drugs in the undergraduate pharmacology practical curriculum.’[2] I have to submit the following in reply:

The usual recommended dose of tinidazole is 2 g/day for three days. The dose (1 g) given in the above letter was inadvertently typed and should be read as 2 g/day. However, the cost factor and the suitability remain the same. Metronidazole is usually given as 800 mg TDS for 5–10 days. Both metronidazole and tinidazole are equally effective, but tinidazole is given for a shorter course and is better tolerated.[3]

With regard to matter of the inadequate price search, kindly note that drug prices vary from time to time, but the relatively lower cost of tinidazole treatment should not be undermined. If one chooses the least expensive brands for tinidazole and metronidazole,[4] the cost for one course would be as shown in Table 1.

**Table 1:** Comparative cost of metronidazole and tinidazole

*Drug*	*Cost (Rs)*	*Daily cost (Rs)*	*Cost per course (Rs)*
Metronidazole (800 mg TDS)	6.22 per 10 tablets each of 400 mg	3.70 per day	26.00 for 7 days course
Tinidazole (1 g BD)	7.25 per 2 tablets (1 g each)	7.25 per day	21.75 for 3 days course

It is clear that the cost of tinidazole is somewhat lower than that of metronidazole and it is more convenient to take twice a day for three days only. It is, therefore, reiterated that using the criteria of efficacy, safety, suitability, and affordability, I would select tinidazole as my “P” drug.
